# Oil Sands Contaminants

**DOI:** 10.1289/ehp.1103746

**Published:** 2011-08-01

**Authors:** Steve E. Hrudey

**Affiliations:** Professor Emeritus, Analytical & Environmental Toxicology Division, Faculty of Medicine & Dentistry, University of Alberta, Edmonton, Alberta, Canada, E-mail: steve.hrudey@ualberta.ca

In the article “Alberta’s Oil Sands: Hard Evidence, Missing Data, New Promises,” Weinhold (2011) misrepresented the findings of our Royal Society of Canada report (Gosselin et al. 2010) too often to recount fully here. Despite requesting my review of a draft, *EHP* chose not to correct many errors I identified, raising questions about *EHP*’s editorial bias on this matter. A few examples must suffice.

Apparently determined to find oil sands–related air quality problems beyond the odor issues we highlighted, Weinhold extracted data from our table summarizing 11 years of regional air quality monitoring data (Gosselin et al. 2010) to conclude:

PM_2.5_ exceedances at Fort McKay have been more than double those at the village of Anzac. … As anecdotal evidence of potential particulate matter concerns, a panel commissioned by Environment Canada to evaluate the impacts of oil sands operations referred to the “ubiquitous dust” that was present during their site visits.

Weinhold failed to explain that the Fort McKay site exceeded the 24 hr objective for PM_2.5_ (30 µg/m^3^) only nine times in 11 years, compared with Anzac at four times in 11 years. The implication that Fort McKay is suffering from oil sands PM_2.5_ air pollution is inaccurate. Fort McKay is a rural northern community surrounded by oil sands surface mines, with local domestic combustion sources and occasional impact from regional forest fire smoke. Weinhold’s attempt to validate his oil sands–related PM_2.5_ pollution case by referring to anecdotal comments about “ubiquitous dust” near Fort McKay reflects his ignorance about what PM_2.5_ measures, because it does not represent visible “dust.”

Apparently searching for other air quality problems, Weinhold paraphrased our report to state: “There are more than 1,400 known pollutants emitted by oil sands operations.” This was based on an inventory of all possible pollutants for developing air monitoring priorities. Weinhold neglected to include our next sentence: “The majority of the total mass emissions (98%) are made up of only fifteen compounds.” But more important, any trace air contaminant expert can verify that thousands of pollutants can be found in any major urban area given sufficiently sensitive analytical techniques. No jurisdiction has air quality criteria for these countless trace substances. Weinhold’s attempted revelation about oil sands contaminants being ignored lacks any meaningful air quality context.

Weinhold and *EHP* also chose not to correct his statements, which he directly attributed to our report:

Studies have found that many toxics … can occur at higher concentrations downstream of oil sands operations than upstream (in some cases all the way to Lake Athabasca), and some of these are elevated enough to kill fish.

We advised *EHP* that we reported no evidence of higher levels of contaminants persisting to Lake Athabasca, nor did Weinhold’s blanket statement about levels being elevated enough to kill fish accurately reflect our conclusions.

Another example of bias in the article appears in the caption of a photograph showing a Fort Chipewyan woman in a cemetery; the caption mentions our panel finding that evidence did not support a link between cancers in that community and oil sands contaminants, while noting that we recommended additional monitoring, but there is no mention that our additional monitoring proposal was made specifically to deal with community concerns. The caption continues: “That leaves this Fort Chipewyan woman still uncertain over what caused the lung cancer that killed her mother, husband, and 27-year-old nephew between 2006 and 2008.” Using this emotive photo surely stoops below the standards of an unbiased scientific journal even if it had acknowledged the overwhelming cause of lung cancer. Readers need to know that extensive air quality monitoring in Fort Chipewyan has shown consistently excellent air quality, which has been verified by personal exposure studies. Regardless, it is crude sensationalism to imply that the personal tragedy depicted in this photo is relevant to cancer being caused by environmental contaminants.

Clearly, Weinhold is entitled to disagree with our panel’s findings, particularly if he is writing an opinion piece. However, it is totally unacceptable for *EHP* to allow him to misinterpret extracts from our report and represent them in his article as if they were our findings. This is particularly egregious when the editors have been informed before publication of these misinterpretations.

In closing, I am compelled to forewarn any future national academy panel that may communicate with *EHP* having any expectation of it being an unbiased, objective scientific journal. *EHP* has behaved no better than agenda-driven commercial media that seek to spin their points of view regardless of the science.
